# Targeting Ferroptosis With Natural Products for the Treatment of Skeletal System Disease: An Updated Review

**DOI:** 10.1111/jcmm.71106

**Published:** 2026-04-02

**Authors:** Qian Yi, Jinye Li, Guotian Luo, Jiachen Li, Dahang Yang, Wei Sun, Weichao Sun, Wei You, Lei Yang

**Affiliations:** ^1^ Department of Physiology, School of Basic Medical Sciences Southwest Medical University Luzhou Sichuan China; ^2^ Department of Spine Surgery Shenzhen Second People's Hospital/First Affiliated Hospital of Shenzhen University Shenzhen Guangdong China; ^3^ Department of Orthopedics Shenzhen Second People's Hospital/First Affiliated Hospital of Shenzhen University Shenzhen Guangdong China; ^4^ Medical Innovation Technology Transformation Center of Shenzhen Second People's Hospital Shenzhen Guangdong China; ^5^ Shantou University Medical College Shantou China

**Keywords:** Ferroptosis, natural products, osteoarthritis, osteoporosis, skeletal system disease

## Abstract

Skeletal system diseases, encompassing chronic disorders of the skeletal system which commonly include osteoarthritis, rheumatoid arthritis, osteoporosis and osteosarcoma. Their incidence rates have been increasing in recent years, resulting in significant social and economic burdens. However, their pathogenesis remains inadequately explored. Ferroptosis, a recently identified form of regulated cell death, differs from other cell death mechanisms, such as apoptosis and autophagy, by primarily involving iron metabolism and lipid peroxidation. The underlying mechanism of ferroptosis is characterised by intracellular iron overload and accumulation of ROS, both of which contribute to the onset of osteoarthritis, rheumatoid arthritis and osteoporosis and are closely linked to the malignancy of osteosarcoma. To enhance understanding of ferroptosis' potential role in the pathophysiology and treatment of skeletal system diseases, this review examines its relationship with these conditions, the mechanisms involved and the therapeutic potential of natural compounds in modulating ferroptosis. By investigating the contribution of ferroptosis to the occurrence and progression of these diseases, novel clinical targets for diagnosis and treatment are proposed.

**Abbreviations**
AA/PC: anemarrhena asphodeloides Bunge/Phellodendron chinense C.K. Schneid
ACC: acetyl‐CoA carboxylase
ACSL4: acyl‐coenzyme A synthetase long‐chain family member 4
AKT: protein kinase B
ALOX15: arachidonate 15‐lipoxygenase
AMPK: AMP‐activated protein kinase
CoA: acyl‐coenzyme A
Cys: cystine
DMT1: divalent metal transporter 1
FLS: fibroblast‐like synoviocytes
FSP1: ferroptosis suppressor protein 1
FTH1: ferritin heavy chain 1
GCL: glutamate‐cysteine ligase
Glu: glutamate
GPX4: glutathione peroxidase 4
GPXs: glutathione peroxidase
GSH: glutathione
H_2_O_2_
: hydrogen peroxide
hBMSCs: human bone marrow‐derived mesenchymal stem cells
HCC: hepatocellular carcinoma
IGF2BP1: insulin‐like growth factor 2 mRNA‐binding protein 1
LPCAT3: lysophosphatidylcholine acyltransferase 3
m6A: N6‐methyladenosine
MDA: malondialdehyde
mTOR: mammalian target of rapamycin
NCOA4: nuclear receptor coactivator 4
Nrf2: nuclear factor erythroid 2‐related factor 2
NSCLC: non‐small‐cell lung cancer
OA: osteoarthritis
OB: osteoblast
OC: osteoclast
OP: osteoporosis
OS: osteosarcoma
PCD: programmed cell death
PI3K: phosphatidylinositol‐3 kinase
PL: phospholipid
PUFA: polyunsaturated fatty acyl
RA: rheumatoid arthritis
RCC: renal cell carcinoma
ROS: reactive oxygen species
SLC3A2: solute carrier family 3 member 2
SLC7A11: solute carrier family 7 member 11
SSD: Skeletal system disease
System XC: cystine/glutamate reverse transporter
TF: transferrin
TFR1: transferrin receptor 1

## 1 Introduction

Skeletal system disease (SSD) refers to a group of chronic disorders affecting the skeletal system which commonly includes osteoarthritis (OA), rheumatoid arthritis (RA), osteoporosis (OP) and others [1, 2]. They are characterised by inflammation, bone or cartilage destruction, fractures, limitation of movement and disability [3, 4]. Osteosarcoma (OS) represents the most aggressive malignancy associated with the skeletal system [5]. The incidence of SSD is increasing in recent years, with RA incidence increasing from 0.5% to 1% [6], OA affecting 10% of men and 18% of women over 60 worldwide [7], and approximately 33% of women and 20% of men will experience fractures caused by OP over the 50 years old population worldwide [8, 9]. SSDs represent a significant global health and economic burden, but their pathogenesis remains unsystematically discussed, and effective therapeutic options for patients are limited. Thus, it is imperative to explore and understand the molecular mechanism of SSD, and more efforts are needed to develop new therapeutic strategies.

Ferroptosis is a newly identified type of programmed cell death (PCD) dependent on iron and is characterised by the accumulation of lipid peroxides and cell membrane rupture [10]. The primary mechanism of ferroptosis is associated with the accumulation of reactive oxygen species (ROS) [11], which has been reported to contribute to the initiation of OA [12] and is involved in the pathological processes of RA [13], OP [14] and OS [15]. Despite numerous studies exploring the relationship between ferroptosis and SSD, the underlying molecular mechanisms remain unclear. Furthermore, therapeutic approaches targeting ferroptosis are emerging as promising avenues for SSD therapy [16, 17]. Notably, ferroptosis regulators derived from natural products have been discovered to show significant effectiveness and safety [18, 19], providing a novel strategy for the prevention and treatment of SDD.

Therefore, in the current review, we systematically summarise the current knowledge about ferroptosis in the pathogenesis of SSD, and discuss the potential benefits of natural products in regulating ferroptosis for SSD prevention and therapy, providing a foundation for future research.

## 2 Ferroptosis

The discovery that erastin and Ras‐selective lethal compounds induced cell death without caspase 3 activation and nuclear morphological changes was found by Dolma et al. in 2003 and Yang et al. in 2008 [20, 21]. This phenomenon significantly differed from apoptosis, autophagy, necrosis and pyroptosis. Until 2012, the term ‘ferroptosis' was first coined by Dixon et al. [22] to define this erastin‐induced cell death. Unlike other types of PCD, such as necrosis, which is also characterised by plasma membrane rupture, ferroptosis is distinguished by cell membrane rupture driven by iron‐dependent ROS accumulation and lipid peroxidation. The ferroptosis cells morphologically exhibit distinctive mitochondrial structural abnormalities, including smaller mitochondria, reduced mitochondrial size, loss of cristae and increased mitochondrial membrane density. During ferroptosis, excessive accumulation of intracellular iron, ROS and lipid peroxides leads to the loss of plasma membrane selective permeability, cell membrane rupture, efflux of cellular contents and ultimately cell death. Since it was discovered, ferroptosis has been explored to play important functions in various diseases, including cancers, neurological disorders, cardiovascular diseases, kidney diseases and musculoskeletal diseases [23, 24, 25, 26].

### 2.1 Ferroptosis Activation and Inhibition

**FIGURE 1:**
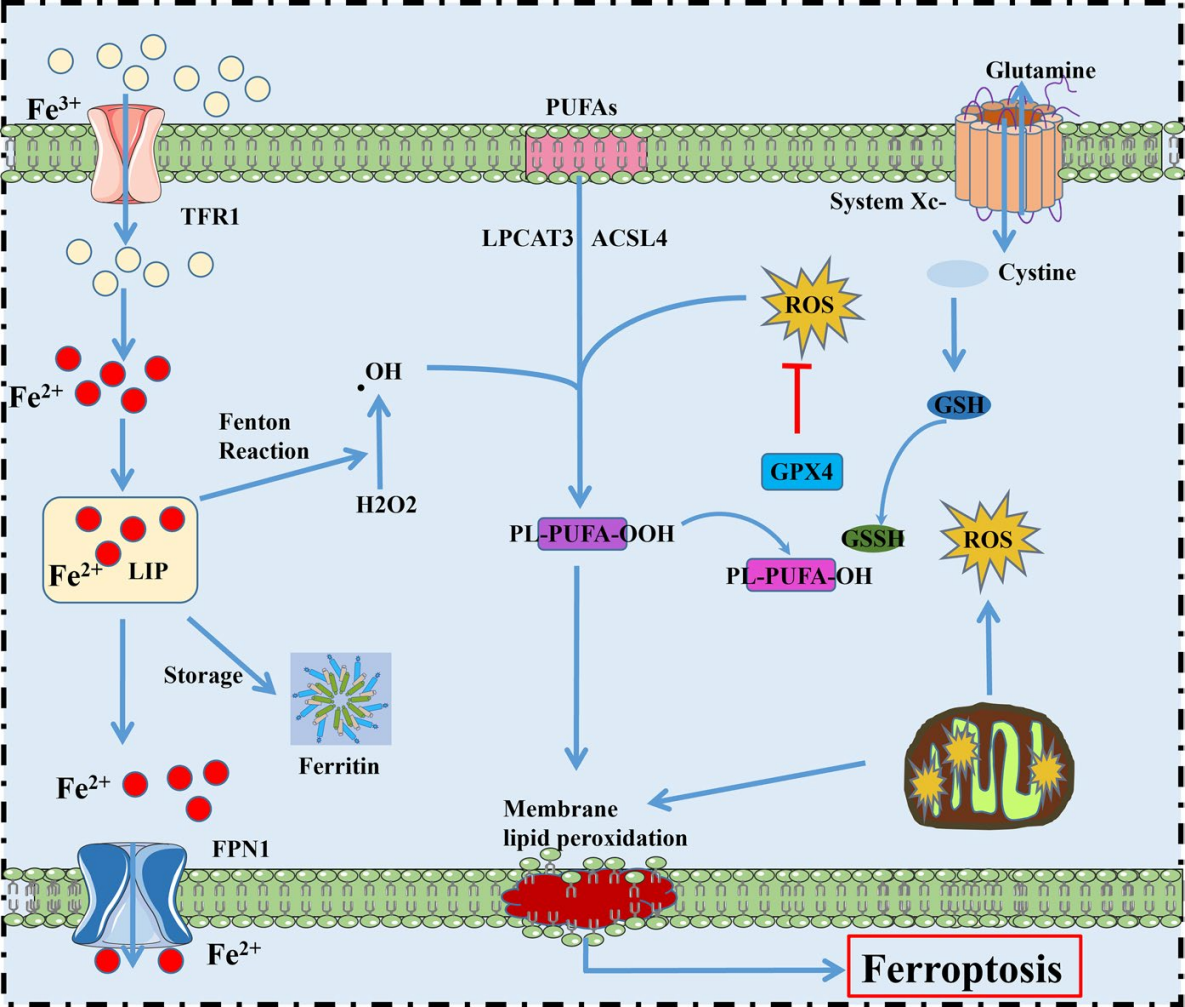
Overview of cellular ferroptosis. TFR and FPN1 regulate intracellular iron Fe^2+^ levels; Ferritin heavy chain 1 (FTH1) binds with Fe^2+^, generating ferritin with iron storage; Fe^2+^ and H_2_O_2_ generate hydroxyl radicals via the Fenton reaction; hydroxyl radicals initiate the peroxidation of lipids, leading to further disruption of cell membrane integrity and ferroptosis; system xc‐ is responsible for cystine transport, and the glutamate‐cysteine ligase (GCL) transforms intracellular cystine into GSH; GSH assists GPX4 in reducing oxidation products and provides substrate for the reduction reaction.

Iron metabolism and lipid peroxidation are key mediators of ferroptosis (Figure 1). Ferroptosis, as indicated by its name, is iron‐dependent, with intracellular iron overload being essential for its occurrence [27]. Normally, human body obtained iron primarily through heme iron and non‐heme iron sources, and non‐heme iron can be derived from either iron ions or ferritin [28, 29]. Transferrin (TF) banded with Fe^3+^, and transported Fe^3+^ into cells via its receptor transferrin receptor (TFR1) [30, 31]. Fe^2+^ enters cells via divalent metal transporter 1 (DMT1) or through ferritin receptor in the ferritin form [32]. And FPN1 and its regulator hepcidin contributed to the iron export from cells [33]. Within cells, ferrireductases mediate the transformation of Fe^3+^ to Fe^2+^, which is either utilised in various biological processes or stored in ferritin [34]. Under normal physiological conditions, the concentration of intracellular free ionic iron remains stable owing to the regulatory effects of ferritin and TFs. While, when the intracellular iron overload, intracellular Fe^2+^ excess leads to the Fenton reaction, which Fe^2+^ catalysed hydrogen peroxide, producing toxic hydroxyl radicals, thereby inducing cellular lipid peroxidation and cellular damage [35].

Lipid peroxidation is another crucial process in ferroptosis. Under normal conditions, polyunsaturated fatty acyl (PUFA) combine with phospholipids (PLs) to form PUFA‐PLs, which regulate the fluidity of cell membranes and maintain the normal physiological function of cells [36, 37]. Lysophosphatidylcholine acyltransferase 3 and acyl‐coenzyme A synthetase long‐chain family member 4 (ACSL4) are the vital enzymes governing PUFA‐PLs production [38]. The Fenton reaction generates toxic hydroxyl radicals and ROS, which promote the formation of PUFA‐PL‐OOH and lipid peroxidation, thereby disrupting the integrity of the cell membrane and leading to ferroptosis [39, 40].

Lipid peroxidation can be neutralised in time to prevent overload and inhibit ferroptosis. Glutathione peroxidase 4 (GPX4), a member of the glutathione peroxidase family, catalyses the conversion of certain lipid hydroperoxides to lipid alcohols, thereby reducing the generation of PL‐OOHs [41]. This process is glutathione (GSH)‐dependent, and is inhibited when GSH biosynthesis is reduced [42]. Furthermore, intracellular GSH biosynthesis requires glutamate, glycine, and cysteine and is reliant on the cystine/glutamate reverse transporter (System XC‐), which influences cysteine content by exchanging extracellular cystine for intracellular glutamate [43, 44]. The system is composed of the light chain subunit solute carrier family 7 member 11 (SLC7A11) and the heavy chain subunit SLC3A2 [45]. The GSH‐dependent detoxification of PL‐OOH by GPX4 prevents lipid peroxidation and ferroptosis, with the SLC7A11‐GSH‐GPX4 axis serving as the primary defense system against ferroptosis.

### 2.2 Signalling Pathways Regulate Ferroptosis

#### 2.2.1 Keap1‐Nrf2‐HO‐1 Pathway

The Keap1‐Nrf2‐HO‐1 pathway plays a crucial role in cellular antioxidant regulation. The negative regulator Keap1 will release Nrf2 under oxidative stress conditions; the released Nrf2 trans‐located to the nucleus and regulated the activation of downstream antioxidant pathways. Firstly, Nrf2 maintained iron homeostasis by controlling HERC2‐ and VAMP8‐mediated ferritinophagy and ferroptosis [46]. Moreover, it has been reported that Nrf2 promoted the transcription of SLC7A11 and the synthesis of GSH [47, 48]. Ferritin regulates intracellular free iron concentration; studies reported that Nrf2 suppresses ferroptosis via inducing the expression of FTH1 [49, 50]. Furthermore, Nrf2 could also attenuate ferroptosis by inducing GPX4 expression [51, 52].

#### 2.2.2 AMPK Signalling Pathway

AMP‐activated protein kinase (AMPK) is a key regulator of cellular energy balance and plays a significant role in maintaining cell physiological function. It also plays an important role in mitochondrial biosynthesis, dynamics and mitophagy. Recently, AMPK was also reported to be involved in the regulation of ferroptosis, both promoting and inhibiting its occurrence. For example, activated AMPK increases mitochondrial biogenesis and mitophagy, promoting mitochondrial homeostasis, thereby reducing ROS levels and inhibiting ferroptosis [53]. Moreover, AMPK negatively regulates fatty acid synthesis by inhibiting the phosphorylation of acetyl‐CoA carboxylase, which reduces the availability of PUFAs for lipid peroxidation and suppresses ferroptosis [54].

However, activation of the AMPK/mTOR pathway has also been shown to inhibit GPX4 expression and promote the occurrence of autophagy‐dependent ferroptosis [55]. ROS‐activated AMPK suppresses mTORC1 function, induces lysosomal degradation of xCT, and ultimately triggers ferroptosis [56]. Additionally, activated AMPK promotes the formation of the BECN1‐xCT complex, which inhibits the activity of xCT to reduce GPX4 expression, thereby inducing ferroptosis [57]. The relationship between AMPK and ferroptosis is complex; further studies are needed to explore and clarify AMPK's specific role in ferroptosis regulation.

#### 2.2.3 PI3K‐Akt–mTOR Pathway

The PI3K‐Akt–mTOR pathway plays a crucial role in cell proliferation, apoptosis and survival. Recent studies have highlighted its aberrant activation as a contributing factor to ferroptosis. It has been reported that activated‐PI3K/AKT/mTOR suppressed ferroptosis through SREBP1/SCD1‐mediated lipogenesis [58, 59]. Moreover, this pathway positively modulated the expression of SLC40A1, enhanced intracellular iron outflow, and then suppressed ferroptosis [60]. Furthermore, Cheng et al. [61] demonstrated that this signalling increased the expression of GPX4 and suppressed ferroptosis. The increased phosphorylation of PI3K/AKT/MDM2 reduced p53 accumulation and induced the upregulation of SLC7A11 and GPX4 [62]. In addition, the PI3K/AKT pathway increased HIF‐1α and SLC7A11 expression to neutralise lipid peroxidation and protect against ferroptosis [63].

#### 2.2.4 P53‐Related Pathway

P53, a well‐known tumour suppressor gene, plays a pivotal role in regulating cell survival under stress. Studies have revealed that p53 can induce cell death via inhibiting the expression of its target gene SLC7A11, thereby limiting the synthesis of intracellular GSH and promoting ferroptosis [64, 65]. Moreover, mitochondrial p53 binds to SLC25A37, resulting in enhanced iron uptake capacity of SLC25A37, which caused an overload of iron in the mitochondria and ferroptosis [66]. Furthermore, P53 promotes the transcription of TfR1, which facilitates Fe^3+^ entry into cells, thereby increasing iron uptake in cardiac cells and triggering ferroptosis [67]. ALOX15, a member of the lipoxygenase family that aggravates cellular lipid peroxidation and causes ferroptosis, p53 contributed to ferroptosis via regulating ALOX15 expression [68, 69]. Furthermore, Chu et al. [70] reported that ALOX12 is critical for p53‐mediated ferroptosis. Recent study has shown that P21 inhibits ferroptosis by interacting with Nrf2, Yang et al. [71] reported that doxorubicin significantly reduces the expression of p53, and then decreases p21 and Nrf2 expression, and promoting ferroptosis.

#### 2.2.5 Hippo‐YAP/TAZ‐Related Pathway

**FIGURE 2:**
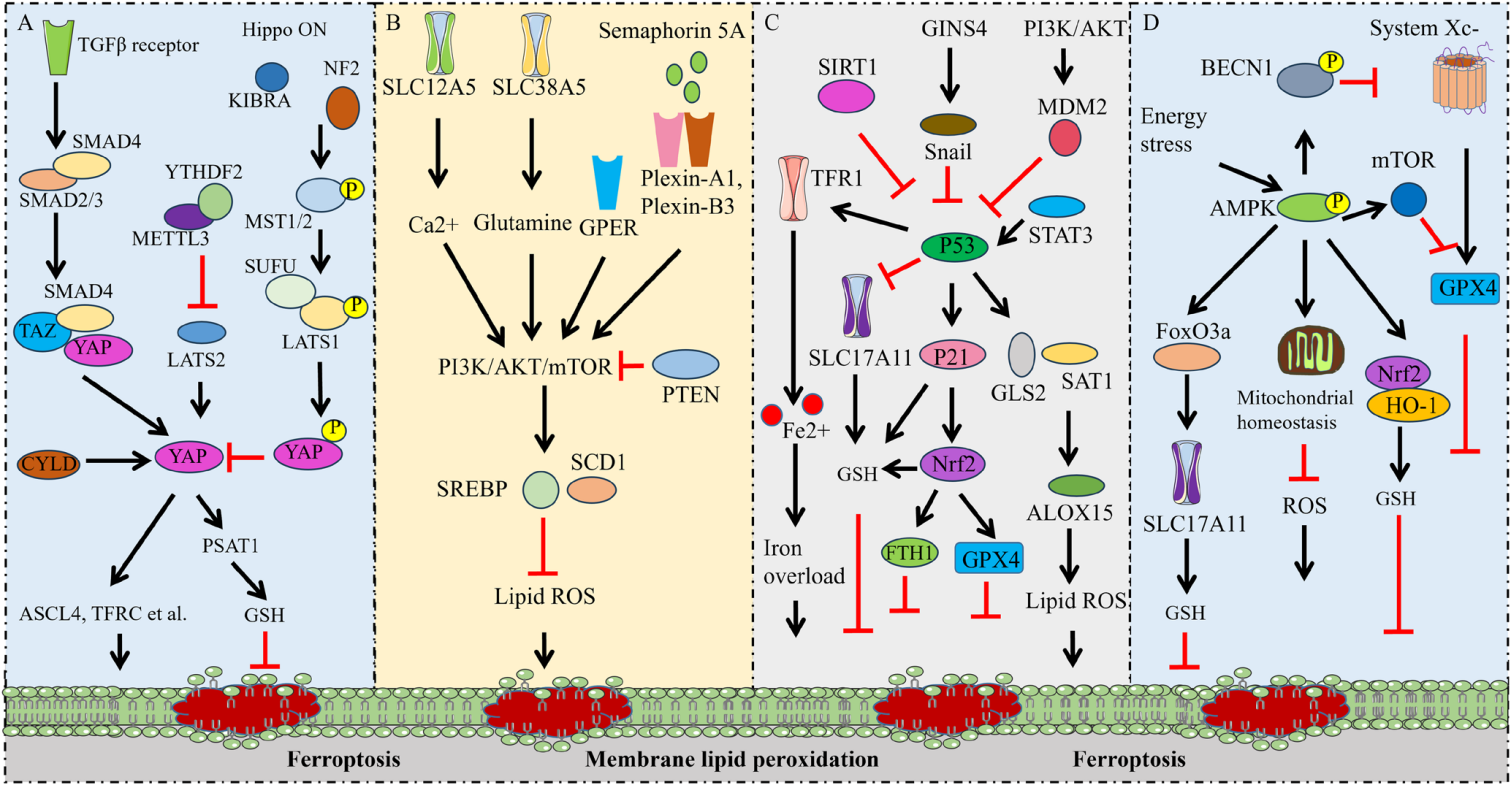
Key regulatory signalling pathways of ferroptosis. (A) the regulatory mechanism of Hippo‐YAP/TAZ signalling in ferroptosis. (B) the regulatory mechanism of PI3K/AKT/mTOR signalling in ferroptosis. (C) the regulatory mechanism of p53 signalling in ferroptosis. (D) the regulatory mechanism of AMPK signalling in ferroptosis.

The Hippo‐YAP/TAZ pathway plays a critical role in the regulation of cell fate, tissue growth and regeneration by responding to extracellular stimulus including cell adhesion, cell density and matrix stiffness. It has been found that cells grown with high density are often more resistant to ferroptosis induced by both cysteine deprivation and GPX4 inhibition, suggesting the involvement of the Hippo pathway in ferroptosis regulation [72]. Hippo/YAP signalling modulates cell ferroptosis and prostate cancer progression via increasing the expression of ACSL4 and TFRC [73]. Moreover, YAP/TAZ induces the expression of SLC7A11, thus enabling hepatocellular carcinoma cells to overcome Sorafenib‐induced ferroptosis [74]. The cell density regulated ferroptosis is mediated by TAZ through the regulation of EMP1‐NOX4 in renal cell carcinoma [75]. In addition, YAP/TAZ contributed ferroptosis resistance in non‐small‐cell lung cancer cells by upregulating the expression of FTH1 and FTL1 [76]. Figure 2 summarises the signalling pathways involved in ferroptosis regulation.

## 3 Ferroptosis and Skeletal System Disease

### 3.1 Ferroptosis and OA

Osteoarthritis is the most common degenerative joint disorder, characterised by symptoms such as joint pain, stiffness, limited mobility and deformity. Its prevalence increases with age, and the incidence rate is 10% in men and 18% in women over 60 years old worldwide [7]. The pathological hallmarks of OA include articular cartilage loss, sclerosis of the subchondral bone, formation of osteophytes and synovitis [77].

Ferroptosis has been implicated in the progression of OA. Elevated iron levels have been observed in the joints of patients with OA [78]. In 2020, Guo group's study first reported that ferric ammonium citrate could induce iron overload in chondrocytes, promoting ROS production and accumulation and ferroptosis [79]. Moreover, excessive stress loading activates the Piezo1 channel, leading to increased calcium influx and accumulation of calcium ions, and then impaired GSH production, reduced GPX4 levels, leading to ferroptosis in chondrocytes [80]. Furthermore, IL‐1β enhances the expression of ACSL4, p53 and TfR1, while inhibiting the expression of GPX4 and SLC7A11, resulting in iron overload, oxidative stress and ferroptosis [81]. IGF2BP1 promoted Fe^2+^ accumulation, ROS production and bolstered chondrocytes ferroptosis of OA by targeting the m6A/MMP3 axis [82]. In addition, SCP2 transports cytoplasmic lipid hydroperoxides to the mitochondria, resulting in mitochondrial membrane damage and ROS release, thus accelerating chondrocyte ferroptosis [83].

**FIGURE 3:**
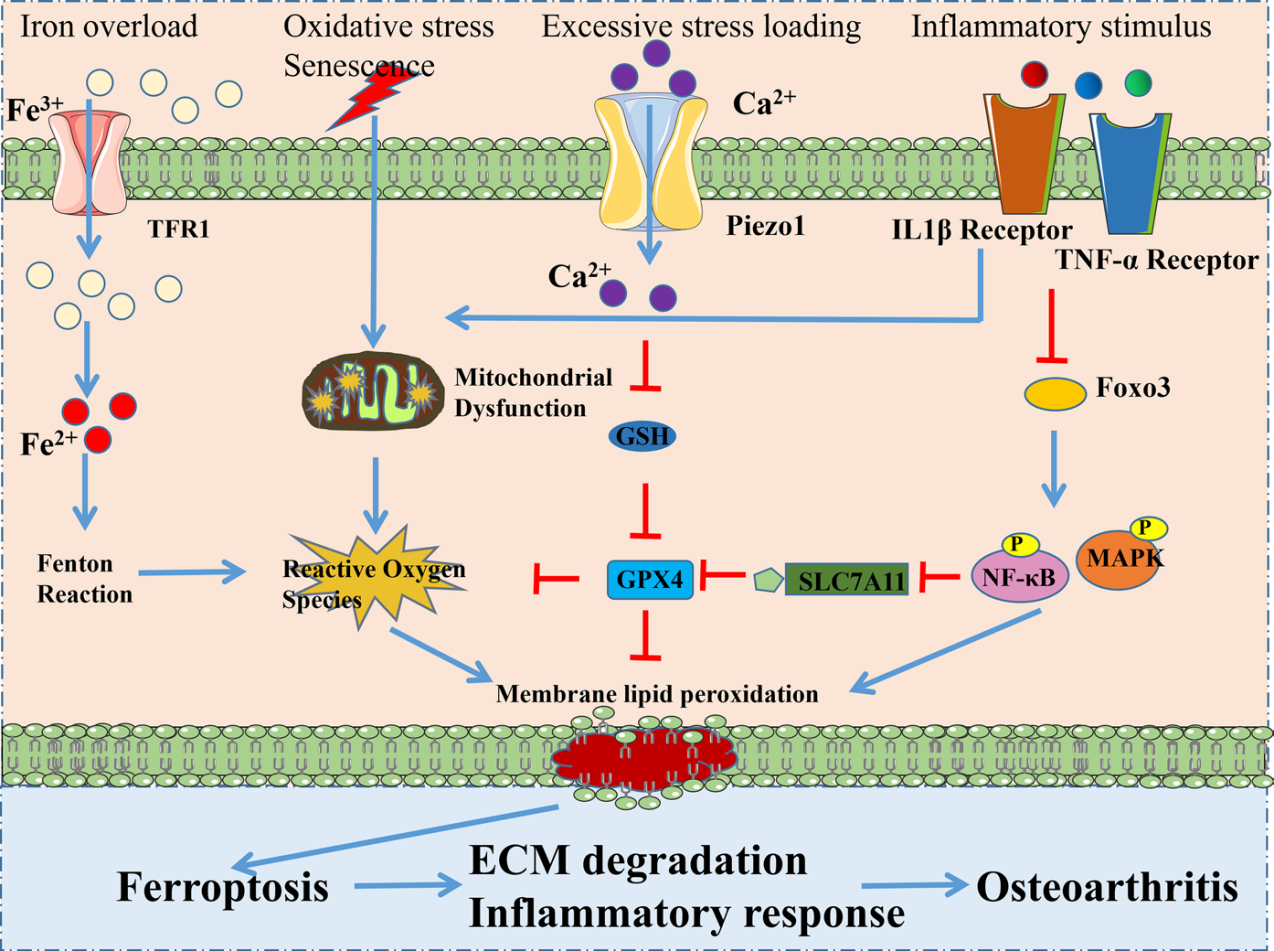
Mechanisms and key regulatory signalling pathways of ferroptosis in chondrocytes. Iron overload increases the intracellular Fe^2+^ concentration and induces ferroptosis. Oxidative stress or senescence causes mitochondrial dysfunction and ROS release. Excessive stress loading or inflammatory stimuli influence the activation of the GPXS/SLC7A11 signalling pathway. These stimuli promote chondrocyte ferroptosis, leading to extracellular matrix degradation, inflammatory response and ultimately osteoarthritis progression.

Synovitis is a significant feature of OA. Ma et al. [84] reported that GPX4 and GSH were downregulated, while ferrous ions were upregulated in synovial tissue samples from patients with OA. Moreover, in LPS‐induced synovitis, MDA levels and iron content were elevated and GPX levels were reduced; icariin protected synoviocytes via the inhibition of ferroptosis by activating the Xc‐/GPX4 axis [85]. Knee OA synoviocytes undergo ferroptosis, contributing to the chondrocyte's matrix degradation and neuropathic pain [86]. The mechanisms and key regulatory signalling pathways of ferroptosis in OA are illustrated in Figure 3.

Given the important regulator role of ferroptosis in OA, targeting ferroptosis may be a novel therapeutic strategy for OA treatment. Deferoxamine, an effective iron‐chelating agent, alleviated OA by inhibiting chondrocyte ferroptosis [87]. Moreover, adenovirus‐mediated expression of FTH1 inhibited the MAPK pathway and ferroptosis in chondrocytes, and suppressed extracellular matrix degradation [88]. MSC‐derived exosomes prevented macrophage ferroptosis via inducing expression of GOT1/CCR2, and then rescued cartilage injury in OA [89]. In addition, Han et al. [90] reported that moderate mechanical stress could activate the Nrf2 antioxidant system, inhibited the NF‐κB signalling pathway, and suppressed chondrocyte ferroptosis and cartilage matrix degradation by regulating P53, SLC7A11 and GPX4.

### 3.2 Ferroptosis and RA

RA is a multifactorial autoimmune disease of unknown aetiology, primarily characterised by inflammatory responses, joint pain and progressive cartilage destruction. It has been reported that iron metabolism is different in RA than in general health and focal iron overload is frequently observed in RA patients [91, 92]. Recently, Yang et al. found that the expressions of GSH, GPX4, Nrf2 and Keap‐1 were lower, and the ferritin was higher in RA patients. Inhibition of oxidative stress‐induced ferroptosis can alleviate RA symptoms in humans. Moreover, excess iron in synovial fluid positively correlates with RA disease severity and aggravates arthritis by inducing macrophage ferroptosis [93]. SIRT1 has been proved downregulated in RA and its function in inhibiting the ferroptosis of synoviocytes has also been suppressed [94]. LPS triggered ferroptosis via NCOA4‐mediated ferritinophagy in RA fibroblast‐like synoviocytes (FLSs) and induced inflammation under hypoxic conditions [95]. Furthermore, CD8^+^ T cells deliver IFNγ to RA‐FLS, regulating the GPX4 antioxidant signalling pathway to promote ferroptosis and inhibit the viability of RA‐FLS [96].

However, some studies reported contradictory findings. For example, ferroptosis has been found to decrease in the RA synovium and FLS compared with healthy controls [97]. Moreover, Wu et al. [98] found that TNF antagonists induced ferroptosis in fibroblasts, thereby attenuating arthritis progression in a collagen‐induced arthritis model. Recently, Cathepsin B has been found highly expressed in patients with RA, its inhibitor reduced RA‐FLS proliferation and migration through leading the ferroptosis in RA‐FLS, targeting ferroptosis may be a potential treatment for RA [99]. In addition, Ruan et al. [100] reported that iron released from Fe_3_O_4_ nanoparticles could induce ferroptosis in both resident inflammatory cells and proliferating FLSs, presenting a novel therapeutic approach for RA.

These conflicting results may be due to the multifactorial nature of RA, where dysregulation in FLSs, dendritic cells, chondrocytes, macrophages and lymphocytes contributed to the progression of RA. The impact of ferroptosis in RA may vary depending on the specific cell type undergoing ferroptosis. Therefore, further clinical data and experimental evidence are necessary to explore the relationship between ferroptosis and the development of RA in future.

### 3.3 Ferroptosis and OP

Osteoporosis is a prevalent metabolic bone disease, with osteocytes, osteoblasts, osteoclasts and mesenchymal stem cells involved in its pathological process. Under normal conditions, the homeostasis of bone‐forming and bone‐resorbing is maintained by the balanced activity of osteoblasts and osteoclasts. In OP, the bone homeostasis is broken, resulting in decreased bone mass, increased bone brittleness and fracture risk. Numerous studies indicate an association between ferroptosis of bone cells and OP. For example, iron overload leads to excessive free Fe^2+^, initiating the Fenton reaction and producing a substantial amount of ROS. These ROS activate a series of intracellular signalling pathways, which promote bone resorption and inhibit bone formation, thereby causing OP [101]. Osteocyte ferroptosis, induced by ATF3/TFR1, plays a role in cortical bone loss during ageing [102]. Yang et al. [103] reported that targeting ferroptosis suppresses osteocyte glucolipotoxicity and alleviates diabetic OP. Similarly, Fu et al. [104] revealed that eldecalcitol ameliorates osteocyte senescence and associated ferroptosis, contributing to bone preservation in OP.

Osteoblastic ferroptosis has also been shown to contribute significantly to OP pathogenesis. Osteoporotic bone loss from excess iron accumulation is driven by NOX4‐triggered ferroptosis in osteoblasts [105]. Epigenetic suppression of GPX4 through DNMT aberration induced osteoblastic ferroptosis and exacerbated OP [106]. Inhibiting osteoblast ferroptosis can suppress OP. YBX1 could alleviate ferroptosis via the ATF4/FSP1 axis in osteoblasts, suggesting that YBX1 could be a potential therapeutic target for OP treatment [107]. Moreover, mangiferin attenuates OP by inhibiting osteoblast ferroptosis through the Keap1/Nrf2/SLC7A11/GPX4 pathway [108]. ED‐71 ameliorates bone regeneration in type 2 diabetes by reducing osteoblast ferroptosis through the HIF1α pathway [109].

In contrast, some studies report that osteoclast ferroptosis is suppressed, and osteoclastogenesis is enhanced in OP. For example, reduced IRF9 expression in OP leads to overactive osteoclastogenesis and decreased osteoclasts ferroptosis [110]. Inducing ferroptosis in osteoclasts and suppressing osteoclast viability may present a potential strategy for OP. P. Ga‐containing calcium titanate layer has been shown to inhibit osteoclastogenesis by inducing osteoclast ferroptosis and promoting the differentiation of hMSCs into osteoblasts [111]. Moreover, Jin et al. [112] demonstrated that artesunate induces osteoclast ferroptosis, inhibits osteoclast differentiation and prevents iron overload‐induced bone loss. Furthermore, zoledronic acid has been shown to improve OP by inducing osteoclast ferroptosis through the p53 signalling pathway [113].

**FIGURE 4:**
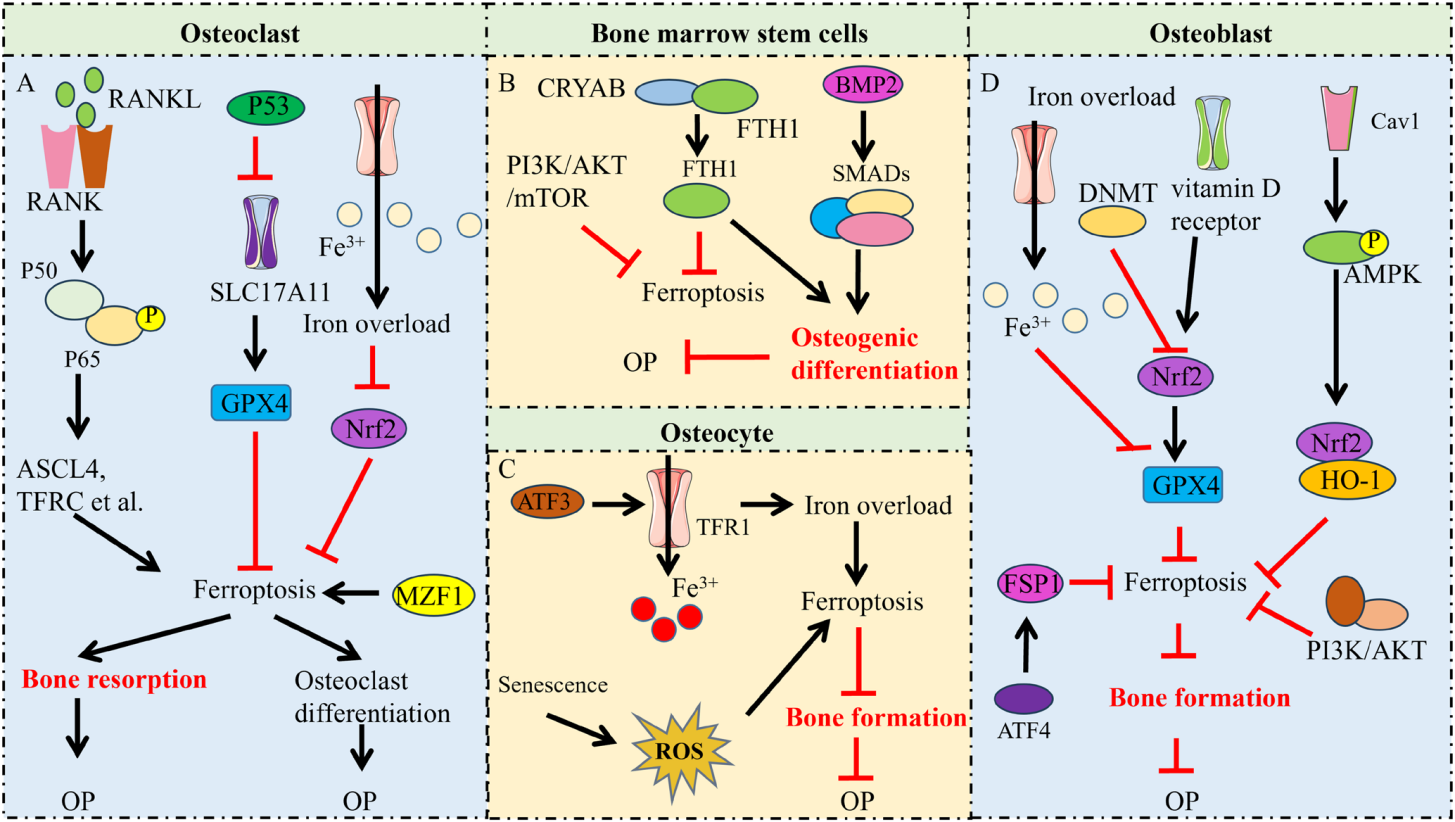
Mechanisms and key regulatory signalling pathways of ferroptosis in OP. (A) the activating of RNAKL signalling pathway and iron overload induces ferroptosis of osteoclasts and thereby promoting bone resorption and OP. (B) PI3K/AKT/mTOR and BMP/SMADs signalling pathway regulates the ferroptosis and osteogenic differentiation of BMSCs, the inhibition of BMSCs ferroptosis help to prevent OP. (C) iron overload and senescence promoting the ferroptosis of osteocytes, their bone formation capacity were inhibition and thereby promoting OP; (D) PI3K/AKT, AMPK and Nrf2/GPX4 signalling pathways regulates the ferroptosis of osteoblasts, the bone formation capacity of osteoblasts undergoing ferroptosis was suppressed and then inducing OP progression.

The osteogenic differentiation of human bone marrow‐derived mesenchymal stem cells (hBMSCs) plays a role in alleviating OP. Iron overload inhibited osteogenic commitment and differentiation of MSCs via the induction of ferritin [114]. Inhibiting ferroptosis in BMSCs can enhance their osteogenic differentiation capacity. CRYAB suppresses ferroptosis and promotes osteogenic differentiation of hBMSCs via binding and stabilising FTH1 [115]. Moreover, aucubin protected against ferroptosis and facilitated osteogenic differentiation of hBMSCs by activating the BMP2/SMADs pathway and attenuating the progression of OP [116]. Furthermore, Poliumoside protects against type 2 diabetes‐related OP by promoting osteogenic differentiation and suppressing BMSC ferroptosis via the Nrf2/GPX4 pathway [117]. Mechanisms and key regulatory signalling pathways of ferroptosis in OP are illustrated in Figure 4.

### 3.4 Ferroptosis and Osteosarcoma

OS is the most prevalent orthopaedic malignancy that originated from mesenchymal cells and is characterised by lung metastasis and resistance to chemotherapy. It has been reported that the absorption rate of iron in OS cells is exceptionally high, suggesting that ferroptosis may play a role in the progression of OS [15]. The dysregulation of ferroptosis related to SLC7A11, GPX4 or oncogenic genes influences ferroptosis in OS. For example, KDM4A mediated histone demethylation of SLC7A11 and then inhibited ferroptosis in OS [118]. Li et al. [119] reported that circRNA‐BLNK inhibited ferroptosis and promoted OS progression by regulating the miR‐188‐3p/GPX4 axis. Moreover, the oncogene PTPRC blocks the TFEB/FTH1 signalling pathway and inhibits ferroptosis in OS [120]. SLC38A5 is upregulated in OS and is associated with poor prognosis in patients; it suppresses ferroptosis via glutamine‐mediated activation of the PI3K/AKT/mTOR signalling pathway [121]. Elzbieta et al. [122] demonstrated that FSP1 is a predictive biomarker of OS cells' susceptibility to ferroptosis, with its expression regulated by p53.

Given the downregulation of ferroptosis in OS, its induction has emerged as a potential therapeutic strategy for OS treatment. For example, ANK1 inhibited the proliferation, migration and invasion of OS cells by promoting ferroptosis [123]. Bone‐targeting exosome nanoparticles activate the Keap1/Nrf2/GPX4 signalling pathway to induce ferroptosis in OS cells and suppress OS progression [124]. In addition, the natural compound oridonin could promote the accumulation of ROS and Fe^2+^ in OS cells, as well as reduce mitochondrial membrane potential, induce ferroptosis and suppress the growth of OS cells [125].

Furthermore, it was well known that cancer cells gradually develop resistance to chemotherapy‐induced cell death, while recent studies show that targeted induction of ferroptosis combined with chemotherapy agents brought new opportunities for OS treatment. For example, NGR (Asn‐Gly‐Arg)‐modified cancer‐associated fibroblasts‐derived exosomes target tumour vasculature to induce ferroptosis and overcome chemoresistance [126]. The induction of ferroptosis by impairing STAT3/Nrf2/GPX4 signalling enhances the sensitivity of OS cells to cisplatin [127]. The reduction of FTH1 translation dramatically increases ferroptosis and promotes the sensitivity of OS cells to chemotherapy drugs [128]. Nanoparticle‐encapsulated doxorubicin alleviates drug resistance in OS via inducing ferroptosis [129]. Inhibitors of APE1 redox and ATM synergistically sensitise OS cells to ionising radiation through ferroptosis induction [130]. In addition, the natural compound, ursolic acid, degraded ferritin by inducing intracellular overload of ferrous ions and leading to ferroptosis, enhancing the DNA‐damaging effect of cisplatin on OS cells [131].

## 4 Natural Products in Regulating Ferroptosis in SSD

Natural products derived from plants and microorganisms have long been utilised in treating human diseases, including cancers, viral diseases, inflammatory‐related diseases [132]. Recently, lots of these compounds were proved to be natural regulators of ferroptosis through the modulation of lipid metabolism, iron metabolism, mitochondrial function or NRF2 signalling [19].

### 4.1 Natural Products in Regulating Ferroptosis in OA

It has been reported that iron homeostasis plays a positive role in maintaining articular cartilage health. Studies have proved that natural compounds could protect chondrocytes via regulating iron metabolism. Biochanin A, an isoflavone isolated from Huangqi, could directly reduce intracellular iron concentration by inhibiting TfR1 and promoting ferroprotein and then prevent the OA progress [133]. Moreover, Ruscogenin was reported to induce the upregulation of Ferritin and SLC7A11, regulate iron metabolism, suppress chondrocyte ferroptosis and then attenuate cartilage destruction [134]. Nrf2 regulates FTL/FTH1 expression to control intracellular free iron concentrations, induces cystine uptake and promotes the synthesis of GSH and GPX4. Gamma‐oryzanol disrupts Keap1‐NRF2 binding and then activates Nrf2 signalling, reducing chondrocyte ferroptosis and mitigating OA [135]. Furthermore, protopine, an isoquinoline alkaloid, protected chondrocytes from ferroptosis by activating the Nrf2 pathway [136]. Sappanone A alleviated OA progression by inhibiting chondrocyte ferroptosis via activating the SIRT1/Nrf2 signalling pathway [137].

GPX4 is the only known glutathione peroxidase responsible for reducing liposomal peroxides. Gossypol acetic acid, a natural phenolic compound, alleviated the ferroptosis of chondrocytes and OA progression by increasing GPX4 expression [138]. What's more, Shang et al. [138] further revealed that Gossypol Acetic Acid alleviates chondrocyte ferroptosis in OA by inhibiting GPX4 methylation. Botulinum toxin A, a macromolecular protein extracted from *Clostridium Botulinum* , could improve mitochondrial function and promote the activation of SLC7A11/GPX4 anti‐ferroptosis system in chondrocytes, thereby attenuating OA [139]. In addition, Vinpocetine protected against OA by inhibiting ferroptosis and extracellular matrix degradation through activation of the Nrf2/GPX4 pathway [140]. And Zhang et al. [141] reported that asperosaponin VI suppresses chondrocyte ferroptosis and ameliorates OA by modulating the Nrf2/GPX4/HO‐1 signalling pathway. Further details on the mechanisms of other natural products regulating ferroptosis in OA are summarised in Table 1.

**TABLE 1:** The role of natural products in ferroptosis in OA.

Ingredients	Functions and mechanisms	References
Paeonol	Suppressed chondrocyte ferroptosis via activation of AMPK/Nrf2/Gpx4 signalling	[142]
Quercetin	Suppressed chondrocyte ferroptosis via activation of AMPK/Nrf2/Gpx4 signalling	[143]
Quercetin	Suppressed chondrocyte ferroptosis via activation of SIRT1/Nrf−2/HO−1 signalling	[144]
Notopterol	Suppressed chondrocyte ferroptosis by modulating the PI3K/Akt/GPX4 axis	[145]
Geniposidic acid	Inhibited inflammation and chondrocyte ferroptosis through activating Nrf2	[146]
D‐mannose	Inhibited chondrocyte ferroptosis by suppressing HIF‐2α and increased Gpx4 and Slc7a11	[147]
Icariin	Inhibited chondrocyte ferroptosis by enhancing the SLC7A11/GPX4 signalling	[148]
Paeoniflorin	Suppressed chondrocyte ferroptosis via the p53/SLC7A11/GPX4 pathway	[149]
EGCG	Inhibited chondrocyte ferroptosis by increasing GPX4, reduced abnormal Fe^2+^ accumulation	[150]
Baicalin	Inhibited chondrocyte ferroptosis by activating Nrf2 antioxidant system	[151]
Sarsasapogenin	Inhibited chondrocyte ferroptosis through the promotion of YAP1	[152]
Kukoamine A	Inhibited chondrocyte inflammation and ferroptosis via the SIRT1/GPX4 signalling pathway	[153]
Brevilin A	Inhibited inflammation and ferroptosis via the SIRT1/Nrf2/GPX4 signalling pathway	[154]
Acetyl zingerone	Inhibited chondrocyte ferroptosis by activating the Nrf2/HO‐1/GPX4 signalling pathway	[155]
Curcumin	Inhibited chondrocyte ferroptosis by activating Nrf2 signalling	[156]
Tanshinone IIA	Inhibited chondrocyte ferroptosis by decreasing iron levels, ROS and MAD, increased GSH	[157]
Cardamonin	Inhibited chondrocyte ferroptosis and mitochondrial morphology by increasing P53 signalling	[158]
Baicalein	Inhibited chondrocyte ferroptosis by improving the activity of AMPK/Nrf2/HO‐1 signalling	[159]
TF3	Inhibited chondrocyte ferroptosis by enhancing Nrf2/Gpx4 signalling pathway	[160]
Astaxanthin	Inhibited chondrocyte ferroptosis by enhancing the SLC7A11/GPX4 signalling	[161]

### 4.2 Natural Products in Regulating Ferroptosis in RA

Chronic pain is the key manifestation of RA. It has been reported that Anemoside B4, the bioactive ingredient of triterpenoid saponins in the traditional Chinese medicine pulsatilla, alleviates RA pain via suppressing ferroptosis‐mediated inflammation [162]. Moreover, the dysregulation of FLS also contributed to the progression of RA. Amentoflavone, a polyphenolic compound derived from Selaginella tamariscina, induced ferroptosis to inhibit the proliferation, invasion and inflammation in RA‐FLS [163]. JinWu JianGu capsules improved RA primarily through the integrated regulation of the SLC7A11/GSH/GPX4 pathway in M1 macrophages and attenuated ferroptosis [164]. Similarly, Jingfang Granules alleviates lipid peroxidation‐induced ferroptosis in RA by modulating gut microbiota, metabolism of short chain fatty acids and AMPK signalling [165]. Wuwei Ganlu and Myricetin alleviate RA by inhibiting M1 macrophage polarisation through modulation of SHBG/SREBP1‐mediated lipid metabolism [166]. Furthermore, Huo et al. [167] found that cupuncture suppressed synovitis of RA by repressing ferroptosis via butyric acid. Osmundacetone reduced RA‐induced swelling and bone destruction, as well as alleviating inflammation‐related factors and oxidative stress via regulating osteoclast ferroptosis [168].

However, some studies present contradictory findings. Sun et al. [169] reported that Asiatic acid induces ferroptosis in RA‐FLS via the Nrf2/HMOX1 pathway to relieve inflammation in RA. And it has been demonstrated that wasp venom could accumulate lipid ROS to induce GPX4‐mediated ferroptosis and were potential therapeutic agents for RA through modulating JAK/STAT signalling pathway [170]. The effects of ferroptosis in RA may depend on the specific cell type undergoing ferroptosis.

### 4.3 Natural Products in Regulating Ferroptosis in OP

OP is a major clinical issue in older individuals, featured with a disorder of decreased bone mass, microarchitectural deterioration and fragility fractures. Due to abnormal osteoblasts and osteoclasts homeostasis, bone‐forming is weakened and bone‐resorbing is enhanced, resulting in decreased bone mass and increased bone brittleness. Therefore, maintaining osteoblast activity and function, and suppressing osteoclast activity are critical strategies for OP treatment. It has been reported recently that ferroptosis is involved in the pathogenesis of OP via regulating the activity of both osteoblasts and osteoclasts [171].

Research showed that osteoblast ferroptosis is a key factor in age‐related OP [172], and inhibiting ferroptosis in osteoblasts may help alleviate the progression of OP. For instance, Proanthocyanidins treatment improves trabecular bone structure, reduces bone marrow adipocytes, decreases oxidative stress and enhances the expression of key osteogenic proteins by regulating the SIRT6/Nrf2/GPX4 pathways [173]. Deng et al. [108] found that Mangiferin attenuated OP by inhibiting osteoblastic ferroptosis via the Keap1/Nrf2/SLC7A11/GPX4 pathway. Similarly, Fructus Ligustri Lucidi protects osteogenic potential by inhibiting ferroptosis through the Nrf2/HO‐1/GPX4 signalling pathway [174]. Moreover, Poliumoside protects against type 2 diabetes‐related OP by suppressing ferroptosis via activation of the Nrf2/GPX4 pathway [117]. Asperosaponin VI reduced osteoblast ferroptosis and alleviated diabetic OP by increasing the GPX4 expression [175]. Picein alleviated oxidative stress and promoted bone regeneration in osteoporotic bone defects by inhibiting ferroptosis through the Nrf2/HO‐1/GPX4 pathway [176].

On the other hand, some natural compounds contributed to bone homeostasis in OP via regulating osteoclastogenesis. It has been reported that artesunate inhibits osteoclast differentiation by inducing ferroptosis and preventing iron overload‐induced bone loss [112]. Moreover, Saikosaponin A promoted osteoclast ferroptosis, attenuated osteoclastogenesis and bone loss by inhibiting the Nrf2/SLC7A11/GPX4 axis [177]. Icariin regulated systemic iron metabolism and promoted osteoclast ferroptosis and thus inhibits bone loss and iron overload‐induced OP [178].

Moreover, Mesenchymal stem cells (MSCs), especially bone marrow mesenchymal stem cells, play a vital role in bone metabolism and tissue repair, with their ability to differentiate into osteoblasts being crucial in the treatment of OP. For example, Crocin can inhibit ferroptosis via the Nrf2/GPX4 pathway and promote the osteogenic function of BMSCs [179]. Li et al. [180] reported that 4‐Octyl Itaconate attenuates postmenopausal OP by inhibiting ferroptosis and enhancing osteogenesis via the Nrf2 pathway. Wang et al. [181] found that Vaccarin not only suppressed erastin‐induced ferroptosis but also enhanced the osteogenic differentiation of BMSCs, thereby alleviating OP.

However, some studies suggest that inhibiting osteoclast ferroptosis may benefit OP treatment. For instance, Xue et al. [182] demonstrated that aconine attenuates osteoclast‐mediated bone resorption and improves OP via inhibiting NF‐κB signalling and osteoclast ferroptosis. A summary of other natural compounds regulating ferroptosis in OP is provided in Table 2.

**TABLE 2:** The role of natural products in ferroptosis in OP.

Ingredients	Function	Mechanisms	References
Picroside II	Suppressed osteoblast ferroptosis	Regulated the YY1/TGFβ1 axis	[183]
Chikusetsusaponin IVa	Suppressed osteoblast ferroptosis	Intervened the GSK3β/NRF2/GPX4 pathway	[184]
Maresin1	Suppressed osteoblast ferroptosis	NRF2 signalling activation	[185]
Xanthohumol	Suppressed osteoblast ferroptosis	Regulated the Akt/GSK3β/Nrf2 pathway	[186]
Qing'e Pill	Suppressed osteoblast ferroptosis	Regulated the PI3K/AKT pathway	[187]
Icariin	Suppressed osteoblast ferroptosis	Activated the antioxidant Nrf2/HO‐1 signalling	[188]
Gastrodin	Suppressed osteoblast ferroptosis	Nrf2 signalling activation	[189]
Resveratrol	Suppressed osteocyte ferroptosis	Regulated the SLC7A11/GPX4 pathway	[190]
Zoledronic acid	Induced osteoclasts ferroptosis	Triggered FBXO9‐mediated p53 degradation	[191]
Angelicin	Suppressed osteoclasts ferroptosis	Activated the Nrf2/HO‐1 signalling	[192]
Sarsasapogenin	Suppressed BMSCs ferroptosis	Restored the GPX4/SLIT3/ROBO1 axis	[193]
Quercetin	Suppressed BMSCs ferroptosis	Inhibited the PI3K/AKT/mTOR pathway	[194]
Astragalus polysaccharide	Suppressed BMSCs ferroptosis	Reduced the accumulation of ROS	[195]
Sarsasapogenin	Suppressed BMSCs ferroptosis	Activated the GPX4/SLIT3/ROBO1 axis	[193]
AA/PC	Suppressed ferroptosis	—	[196]

### 4.4 Natural Products in Regulating Ferroptosis in OS

Various studies have highlighted the potential value of natural products in cancer treatment, including OS. For example, Huang et al. [197] demonstrated that Artesunate exhibits anti‐OS properties by inducing ferroptosis through NCOA4‐mediated ferritinophagy. Shikonin was reported to effectively suppress OS growth with favourable biosafety through regulating the mitochondrial ROS‐regulated HIF‐1α/HO‐1 axis, thereby inducing ferroptosis [198]. Marsdenia tenacissima extract accelerates ferroptosis in OS cells by upregulating HO‐1 and activating mitophagy [199]. Curcumin induces both ferroptosis and apoptosis in OS cells by modulating the Nrf2/GPX4 signalling pathway [200]. Casticin, a natural flavonoid constituent, promotes ferroptosis and inhibits OS growth and metastasis through Fe^2+^ overload and ROS production mediated by HMOX1 and LC3‐NCOA4 [201]. Moreover, Hydroxysafflor yellow A, a natural quinochalcone C‐glycosides compound from *Carthamus tinctorius* L, induces ferroptosis via the HIF‐1α/HK2 and SLC7A11 pathways, suppressing OS progression [202]. Baicalin, a biologically active flavonoid compound isolated from Radix Scutellariae, induces ferroptosis and inhibits OS through a novel Nrf2/xCT/GPX4 regulatory axis [203].

In addition, some compounds could improve the sensitivity of cancer cells to chemotherapy agents. Luo et al. [204] reported that Eriodictyol‐cisplatin coated nanomedicine synergistically promotes ferroptosis and chemosensitivity in OS cells. Formononetin improves cisplatin chemotherapy sensitivity in OS by inducing ferroptosis and reconstructing the immune microenvironment [205]. Shikonin overcomes cisplatin resistance of cancer cells by inducing ferroptosis via upregulation of HMOX1 [206]. Eicosapentaenoic acid enhances the sensitivity of OS to cisplatin by inducing ferroptosis through the NRF2 pathway [207]. Brusatol induces ferroptosis in OS by modulating the Keap1/Nrf2/SLC7A11 signalling pathway, and its combination with doxorubicin (DOX) significantly enhances DOX's anti‐OS efficacy [208]. A summary of other natural compounds regulating ferroptosis in OS is provided in Table 3.

**TABLE 3:** The role of natural products in ferroptosis in OS.

Ingredients	Function	Mechanisms	References
Shikonin	Induced	Promotied Nrf2 ubiquitination and inhibitied the xCT/GPX4 axis	[209]
Bavachin	Induced	Promoted the STAT3/P53/SLC7A11 axis	[210]
Theaflavin‐3,3′‐digallate	Induced	Triggered ROS and MAPK signalling pathways	[211]
Sulforaphane	Induced	Targetied p62 and promoted autolysosomal degradation of SLC7A11	[212]
Gambogenic acid	Induced	Disturbed the redox balance, and activated the P53 signalling	[213]
*Artemisia annua*	Induced	Promoted lipid peroxidation	[214]
Sulfasalazine	Induced	Triggered ferroptosis through the NRF2/SLC7A11/GPX4 signalling axis	[215]
Erianin	Induced	Induced cell ferroptosis and apoptosis	[216]
Capsaicin	Induced	Triggered an increase in intracellular Ca2+ concentration	[217]
Rhizoma Paridis total saponins	Induced	Triggered ferroptosis‐mediated OS suppression through SPI1/LCN2 axis inhibition	[218]
Curculigoside	Induced	Triggered catastrophic buildup of unbound iron and lipid peroxidation	[219]
Vitamin C	Induced	Triggered intracellular ROS‐iron‐calcium signalling crosstalk	[220]
Naringenin	Induced	Increased the accumulation of ROS, iron overload, and MDA	[221]
Curculigoside	Induced	Triggered uncontrolled lipid peroxidation	[219]

## 5 Clinical Studies of Natural Products for the Treatment of SSD

The effectiveness and safety of certain natural products have been partially validated in clinical trials, positioning them as potential therapeutic agents for SSD treatment. For instance, Zhou et al. [156] reported that curcumin can reverse erastin‐induced chondrocyte ferroptosis by upregulating Nrf2. In a randomised controlled trial, a curcuminoid complex combined with diclofenac demonstrated significantly fewer adverse reactions, better tolerability and greater improvements in pain and functional ability compared to diclofenac alone [222]. Moreover, Zeng et al. [223] found that both Curcumin and *Curcuma longa* extract may alleviate symptoms and inflammation in individuals with arthritis, including OA and RA. Crocin has been shown to inhibit ferroptosis via regulating the Nrf2/GPX4 pathway [179], and a clinical trial successful shown that Krocina, a herbal medicine containing crocin, has immunoregulatory effects on patients with OA, improving the disease [224]. Tripterygium wilfordii Hook.f. can regulate ferroptosis via the Nrf2/HO‐1 pathway [225], and it has been widely used for the treatment of RA in China [226]. Moreover, clinical research has shown that Qingre Huoxue Decoction can improve clinical symptoms and relevant indicators in RA patients, with multi‐omics analysis results revealing that its protective mechanisms involve ferroptosis regulation [227]. Various evidence supports that integrating traditional Chinese medicine with Western medicine can improve bone mineral density and reduce pain, and offering a valuable strategy for treating OP [228]. It has been reported that resveratrol can improve osteoporosis in iron‐overloaded mice [229], and a randomised, placebo‐controlled trial confirmed that regular supplementation with resveratrol improves bone mineral density in postmenopausal women [230]. Lycopene, a carotenoid derived from *Lycopersicon esculentum* , has been reported to inhibit mitochondrial damage and ferroptosis [231], and a clinical study demonstrated that the intake of a lycopene‐rich tomato sauce contributed to prevent bone loss in postmenopausal women [232].

## 6 Discussion

While numerous studies highlight the potential of natural products to alleviate symptoms of SSD, significant challenges remain in translating these natural compounds into clinical applications. A primary issue is that many natural compounds have low bioavailability, rapid metabolism and poor absorption. Recently, plant exosome‐like nanovesicles have emerged as effective nanoplatforms for drug delivery, offering a vehicle for active ingredients [233]. For example, exosome‐like nanovesicles derived from Yam can be easily absorbed by osteoblasts, promoting their differentiation and mineralisation, which helps prevent OP [234]. Furthermore, structural modifications of natural compounds can enhance their stability and bioavailability. Zhou et al. [235] demonstrated that chemically modified curcumin (CMC2.24) significantly improved efficacy for OA treatment. In addition, another promising approach is combining natural compounds with clinical biomaterials to address rapid metabolism. Feng et al. [236] reported that platinum nanozyme‐loaded silk fibroin/pullulan hydrogels achieved sustained release for up to 30 days, relieving OA via suppressing ferroptosis.

So far, studies on OS have shown that inducing ferroptosis of tumour cells through natural products can effectively inhibit the malignant progression of the tumour. Research on OA indicated that inhibiting ferroptosis of chondrocytes could maintain their viability and the homeostasis of cartilage. Therefore, natural ferroptosis inducers appear to be a promising strategy for OS treatment, and natural ferroptosis inhibitors benefit OA prevention. However, there exists another critical challenge: that is the complex microenvironment of SSDs, such as the pathological process of RA involves changes in fibroblasts and various immune cells. OP involves the osteoblasts, osteoclasts, immune cells and MSCs. For example, lots of current studies have demonstrated that ferroptosis inhibition is beneficial for RA, and only a few articles have reported the opposite results. Ferroptosis inducers can suppress RA synovial inflammation by inducing ferroptosis in FLSs, but such induction may also impact other cell populations in the synovium, potentially promoting bone erosion and bone destruction [237]. It seems more further studies are needed for a unified conclusion about the opposite results. The essence of OP lies in the dynamic imbalance between osteoblast activity and osteoclast activity. Research results to date indicate that inhibiting ferroptosis in OBs and BMSCs can significantly alleviate bone loss and osteoporosis caused by ageing and oxidative stress. But few studies have shown that ferroptosis induction of OCs also could suppress OP development. In OP, ferroptosis inhibitors may protect osteoblasts and immune cells, thereby enhancing immune responses, but excessive immune activation could lead to overactivation and adverse effects [238]. Balancing ferroptosis induction with osteogenesis and osteoclastogenesis also remains challenging [239]. How to select and when to apply the best inducer or inhibitor applicable to ferroptosis in SSDs requires further in‐depth studies.

To address these challenges, several solutions can be pursued. First, multi‐level studies incorporating genomics, proteomics and metabolomics should be developed to better understand the complexity of ferroptosis. Second, developing cell‐type‐specific ferroptosis regulators will help balance ferroptosis with immune responses and bone metabolism. Third, systematic toxicology and pharmacology studies are essential to assess the safety of natural ferroptosis regulators, facilitating their clinical translation. Moreover, for individual differences, integrate big data information about ferroptosis and patients' situations to carry out precision medicine and personalised treatment.

## 7 Conclusion

Ferroptosis, a novel form of cell death, has become a prominent research focus since it was discovered in 2012. It has been reported that it is involved in the pathogenesis of many diseases, including the skeletal system disease. Various studies have established that ferroptosis plays a significant role in the development of SSDs, such as OP, OA, RA and OS. In this manuscript, we summarise the understanding of the main regulatory mechanisms of ferroptosis and its role in the pathogenesis of SSDs. It also discusses the distinct responses of different cell types, including chondrocytes, OBs, OCs, BMSCs and OS cells to ferroptosis. However, the pathology of SSDs is quite complex, particularly in OA and RA, where the joint microenvironment contains various types of cells, each responding differently to ferroptosis. Therefore, further investigations are required.

Moreover, the great clinical potential of interfering with the ferroptosis pathway, activating or inhibiting, has also been discussed in our article. For cancer treatment, ferroptosis inducers have shown significant therapeutic effects by promoting tumour cell death. However, in RA and OP, ferroptosis inhibitors appear to offer promising results, presenting the challenge of selectively inducing ferroptosis in disease‐affected cells without harming healthy cells. Furthermore, most of the studies to date have been conducted at the animal or in vitro level, with clinical studies still lacking. Furthermore, while natural products have demonstrated significant promise in identifying new active compounds for SSDs, research in this area is still in its early stages and requires further exploration.

In conclusion, ferroptosis is closely linked to SSDs such as OP, OA, RA and osteosarcoma. A deeper understanding of the regulatory mechanisms of ferroptosis, combined with the strategic use of natural compounds as ferroptosis inducers or inhibitors, could provide novel therapeutic targets for the diagnosis and treatment of bone‐related diseases.

## Author Contributions

**Wei Sun:** writing – original draft (equal). **Weichao Sun:** funding acquisition (equal). **Jinye Li:** writing – original draft (equal). **Guotian Luo:** writing – original draft (equal).

## Funding

This research was supported by the Shenzhen‐Hong Kong Jointly Funded Project, Shenzhen Science and Technology Program (SGDX20230116093645007); Shenzhen Medical Research Fund (B2303005); Development and Reform Commission of Shenzhen Municipality (S2002Q84500835); Shenzhen Medical Research Fund (No. A2403030); Joint foundation of Luzhou Government and Southwest Medical University (No. 2024LZXNYDJ104); Shenzhen Science and Technology Projects (No. KCXFZ20230731093059012); Sichuan Science and Technology Program (No. 2026NSFSC0605); Guangdong Basic and Applied Basic ResearchFoundation (NO. 2024B1515120029).

## Ethics Statement

The authors have nothing to report.

## Consent

The authors have nothing to report.

## Conflicts of Interest

The authors declare no conflicts of interest.

## Data Availability

Data sharing not applicable to this article as no datasets were generated or analysed during the current study.
